# Evaluation of Geometric Attractor Structure and Recurrence Analysis in Professional Dancers

**DOI:** 10.3390/e24091310

**Published:** 2022-09-16

**Authors:** Michalina Błażkiewicz

**Affiliations:** Faculty of Rehabilitation, Józef Piłsudski University of Physical Education in Warsaw, 00-809 Warszawa, Poland; michalinablazkiewicz@gmail.com

**Keywords:** attractor reconstruction, recurrence quantification analysis, phase space, dance, pirouette

## Abstract

Background: Human motor systems contain nonlinear features. The purpose of this study was to evaluate the geometric structure of attractors and analyze recurrence in two different pirouettes (jazz and classic) performed by 15 professional dancers. Methods: The kinematics of the body’s center of mass (CoM) and knee of the supporting leg (LKNE) during the pirouette were measured using the Vicon system. A time series of selected points were resampled, normalized, and randomly reordered. Then, every second time series was flipped to be combined with other time series and make a long time series out of the repetitions of a single task. The attractors were reconstructed, and the convex hull volumes (CHV) were counted for the CoM and LKNE for each pirouette in each direction. Recurrence quantification analysis (RQA) was used to extract additional information. Results: The CHVs calculated for the LKNE were significantly lower for the jazz pirouette. All RQA measures had the highest values for LKNE along the mediolateral axis for the jazz pirouette. This result underscores the high determinism, high motion recurrence, and complexity of this maneuver. Conclusions: The findings offer new insight into the evaluation of the approximation of homogeneity in motion control. A high determinism indicates a highly stable and predictive motion trajectory.

## 1. Introduction

According to Stergiou and Decker [1], the deviations observed during multiple repetitions of a given task are defined as movement variability. This variability is an integral feature of living organisms because the repetition of activities is associated with unique neuromotor patterns [2,3]. It implies that even an elite performer cannot perform the same task identically twice. According to Riley and Turvey [4] and van Emmerik et al. [5], human movement systems present stochastic behaviors potentially affected by deterministic and random processes. However, as van Mourik et al. [6] noted, most studies focused only on the repetitive, deterministic features of human motion. Nevertheless, research emphasizing the stochastic aspects of human movement has become more common in recent years. Despite this development, the deterministic and stochastic features of human movement are rarely evaluated together. Accordingly, human motion should be treated as a dynamic system in which a subject moves between states over time.

The dynamical systems approach focuses on how the system (1) maintains its current state (the stability problem), (2) changes or transitions between states (the task of variability and adaptability), and (3) regulates the complexity resulting from interactions between the central nervous system, body, and the surrounding environment. When dealing with dynamic systems, it is worth checking whether they exhibit nonlinear characteristics before applying nonlinear tools [2,7]. Nonlinear tools for evaluating the features of the system mentioned above include the Lyapunov exponent, Hurst exponent, fractal dimension, entropy families [8,9,10], the possibility of attractor reconstruction (phase space reconstruction) [11], and recurrence quantification analysis (RQA) [12]. Kędziorek and Błażkiewicz [13] have shown that sample entropy, fractal dimension, and the Lyapunov exponent are highly applicable to assessing postural control. On the other hand, RQA and the phase space concept in postural control assessment are less common in the literature.

One of the most powerful tools for identifying different behaviors of a dynamical system is the Lyapunov exponent (LyE), more precisely, its value (sign) [14]. It measures the exponential rate at which nearby trajectories converge or diverge. The Lyapunov exponent shows whether the observed dynamic system is stable, periodic, quasi-periodic, or chaotic [15]. Over the years, many algorithms have been developed for calculating Lyapunov exponents from time series [15,16,17,18]. The two most popular are the Wolf algorithm [15] and Rosenstein’s method [16]. According to Rispens et al. [19], the accuracy of Rosenstein’s algorithm is equal to or greater than Wolf’s algorithm. The latter appeared more sensitive to the number of observations and the amount of noise in the data. Thus, Wolf’s algorithm is more accurate for long-term data with low noise levels [16]. In chaos research, another parameter, known as the Hurst exponent, can be defined. The Hurst exponent measures the predictability of a time series and describes its level of statistical self-similarity and long-term memory [20]. The larger the value of H, the smoother the time series. H reaches values between 0 and 1. The properties of H can be summarized as follows: (1) H = 0.5 indicates random series (time series behaves like the Brownian process); (2) 0.5 < H ≤ 1 indicates persistent long-range power-law correlations; (3) 0 ≤ H < 0.5 indicates anti-persistent (short-term memory, anti-correlated) process [21].

The behavior of a dynamic system is typically represented in phase space. A phase space is a space in which all possible states of a system are represented, with each possible state corresponding to one unique point in the phase space. It seems appropriate at this point to mention the definition of an attractor. According to Devaney [22], an attractor is a set toward which most other points from the state space tend toward under iteration. In dimension two, such a set would consist of a collection of limit cycles, equilibrium points, and solutions connecting them. In higher dimensions, these attractors may be much stranger. The dynamics of the attractor itself may be chaotic. There are many possible definitions of chaos. One that is recognized comes from Devaney [22]: Let *X* be a metric space. A continuous map *f*:*X*→*X* is said to be chaotic on *X* if (1) *f* is transitive; (2) the periodic points of *f* are dense in *X*; (3) *f* has sensitive dependence on the initial conditions. An attractor is strange if it displays sensitive dependence on the initial conditions (points initially close to each other on the attractor become exponentially separated with time). According to Takens [23], to reconstruct the state space, two variables are needed: (1) minimal embedding dimension (D) and (2) optimal time delay (lag) − τ (tau) (Table 1).

The embedding dimension is the minimum number of variables required to create a valid state space from a given time series. Vectors in the embedding space (a new space) are constructed from the time-delayed values of the scalar measurement [2]. Many methods (Table 1) have been developed for estimating embedding dimensions [25,26,27,28]. There is no rule for determining the minimum embedding dimension, D, and none of the published proposals are widely accepted. Of all the possible techniques, the correlation dimension and false nearest neighbors stand out the most [29]. The autocorrelation of a time series or mutual information is a common approach for time-lag selection [24]. It is worth noting that both the embedding dimension and the time delay are also necessary when using RQA. RQA quantifies the number and duration of recurrences of a dynamical system represented by its phase space trajectory [12]. RQA allows for a model of features, such as the determinism of a system, the dimensionality or complexity of its dynamics, and the amount of patterning it holds [30]. In this section, it is worth writing that attractor reconstruction and RQA have been successfully used to characterize human behaviors like heart rate variability, postural fluctuations, and the variability and stability of dynamic biological systems [31,32].

Som, et al. [33] proposed high-dimensional shape descriptors for the reconstructed attractors of the center-of-pressure (CoP) paths collected from subjects with Parkinson’s disease to assess their balance impairment. Gates and Dingwell [34] reconstructed a state space for the assessment of shoulder movement. Bradley and Stuart [35] used attractor geometry to describe the predefined motion sequences in dance. Peppoloni et al. [36] characterized the interference of neural control strategies for dynamic fingertip forces from attractor reconstruction. In addition, attractor reconstruction can determine changes in the shape and variability of periodic signals, such as arterial blood pressure signals [37] and electroencephalogram signals [38], providing a two-dimensional attractor with features like density and symmetry [39]. Recurrence quantification analysis was used to determine the structure of CoP data during a standing state [40]. Labini et al. [41] assessed the walking balance complexity of the head, trunk, and pelvis for the gait of normal and hypo vestibular subjects. Riley and Clark [42] examined how the availability of, and alterations in, sensory information influenced the amount, variability, and temporal structure of spontaneous postural sway in young, healthy adults. Moreover, RQA is often used to evaluate heart rate variability [43,44,45]. Until now, no paper has used attractor reconstruction for pirouettes and used RQA to evaluate their repeatability. The purpose of the study was to evaluate the geometric structure of the attractors and analyze the recurrence of two different pirouettes performed by professional dancers.

## 2. Materials and Methods

### 2.1. Participants and Data Collection

A total of 15 modern dance dancers, whose primary preparation was sports acrobatics, participated in this study (Table 2). Participants had no lower limb injuries or balance disorders that would affect the quality of their task performance. It is crucial to mention that the examined dancers were from one dance group and had been dancing with each other for more than four years. The study protocol was approved by the University Research Ethics Committee (SEK 01-09/2020).

**Figure 1:**
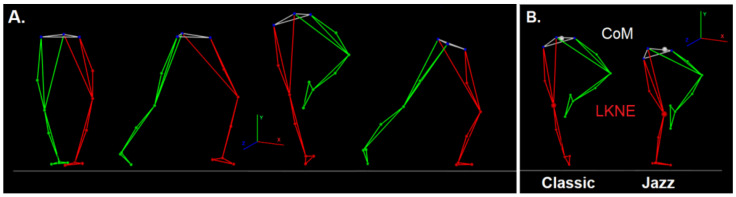
Participant model during a pirouette. (**A**) Motion sequences; (**B**) the location of the center of mass (CoM) and position of the marker on the knee’s lateral epicondyle of the supporting leg (LKNE) for the classic and jazz pirouette. The coordinate system, where: x—horizontal axis (anterior-posterior), y—vertical axis (inferior-superior), and z—horizontal axis (mediolateral).

The kinematic parameters of jazz and classic pirouettes were collected using a three-dimensional motion capture system (Vicon Motion Systems, Oxford, UK). The system consisted of 9 cameras operating at 100 Hz. The two force platforms (Kistler Holding AG, Winterthur, Switzerland) were synchronized with the Vicon system. The platforms operated at 1000 Hz. A total of 15 markers were placed on the subjects, according to the lower body Plug-In-Gait scheme (Figure 1). Each dancer, after warm-up, performed two types of single-turn pirouette *en dehors* (classic and jazz on demi-plié) (Figure 1). All participants were barefoot and performed one turn on the left foot. Dancers rotated all the time in the clockwise direction [46].

### 2.2. Phases of Pirouettes and Parameters

Each pirouette (classic and jazz) begins in the classical fourth position. From this position, the dancer performs plié to initiate a clockwise rotation on the support leg (the forward leg). The turn starts with pushing off the ground with the non-supporting leg and moving the foot up to the knee of the supporting leg. This position remains throughout the rotation. The dancer then pushes up onto the pointe or demi-pointe (the ball of the foot) with the supporting leg and closes both arms in front [46,47,48]. While in the rotation, the dancer maintains their body in the vertical rotational axis. The pirouette ends with a specific body pose, adopted by placing the non-supporting leg on the ground (Figure 1A).

The main difference between the two pirouettes is the range of knee flexion and plantar flexion of the ankle joint of the supporting leg and, thus, the CoM position of the body (Figure 1B). In the classic pirouette, the knee joint should have full extension. The ankle joint should have maximum plantar flexion. In contrast, in the jazz pirouette, the knee joint should remain flexed, and the plantar flexion should be less. Although entire pirouettes were recorded, the phases of turning were taken for analysis. The analyzed movements began with knee flexion, as the first movement of the rotation began and ended when the foot of the non-supporting leg first touched the ground. The three-dimensional CoM and LKNE time series were taken for further analysis (LKNE was the marker on the lateral epicondyle of the left knee).

### 2.3. Phase Space Reconstruction

In this paper, it was decided to assess the structure of the considered time series by checking the nonlinearity, non-stationarity, and signs of chaos, before reconstructing the phase space. All calculations described below were completed using MatLab R2021a software (MathWorks, Natick, MA, USA).

#### 2.3.1. New Time Series Reconstruction

The collected data for both pirouettes, for a single individual, were not long enough to meet the basic requirements for the ability to reconstruct the phase space. A new time series has been reconstructed separately for LKNE and CoM for jazz and classic pirouettes to overcome this issue. The reconstruction steps are below.

(1) The $P_{i}=\left[\begin{array}{ccc} x_{i_{1}} & y_{i_{1}} & z_{i_{1}}\\ \vdots & \vdots & \vdots \\ x_{i_{n}} & y_{i_{n}} & z_{i_{n}} \end{array}\right],$ where $P_{i}$—is the matrix of LKNE or CoM coordinates for the analyzed pirouettes of the *i*-th person (*i* = 1, …, 15); *n*—is the length of the recorded time series. The *n* values were in the range of 101 to 250 frames. The *x*-coordinate describes motion in the anterior-posterior direction, *y*—along a vertical axis (inferior-superior), and *z*—in the mediolateral direction.
(2) The signals from each matrix *P_i_* were resampled to obtain 300 samples. Its new length was longer than the maximum length of the recorded time series (250). The new signals were normalized by their maximal value.
  ${\tilde{P}}_{i}=\left[\begin{array}{c} \begin{array}{ccc} {\tilde{x}}_{i_{1}} & {\tilde{y}}_{i_{1}} & {\tilde{z}}_{i_{1}} \end{array}\\ \begin{array}{ccc} \vdots & \vdots & \vdots \\ {\tilde{x}}_{i_{300}} & {\tilde{y}}_{i_{300}} & {\tilde{z}}_{i_{300}} \end{array} \end{array}\right],$ where ${\tilde{P}}_{i}$—matrix of LKNE or CoM after transformation, for the analyzed pirouettes of the *i*-th person (*i* =1, …, 15).
(3) Next, the new time series for LKNE and CoM for the *x*, *y*, and *z* coordinates were created. Individuals in the new time series have been shuffled to avoid bias based on their order.

$$/shuffle\;in\;the\;matrix/\left[\begin{array}{c} {\tilde{P}}_{1}\\ \vdots \\ {\tilde{P}}_{15} \end{array}\right]=/inversion\;of\;indexes/\lceil \begin{array}{c} \begin{array}{ccc} {\tilde{x}}_{1_{1}} & {\tilde{y}}_{1_{1}} & {\tilde{z}}_{1_{1}}\\ \vdots & \vdots & \vdots \end{array}\\ \begin{array}{ccc} {\tilde{x}}_{1_{300}} & {\tilde{y}}_{1_{300}} & {\tilde{z}}_{1_{300}}\\ {\tilde{x}}_{2_{300}} & {\tilde{y}}_{2_{300}} & {\tilde{z}}_{2_{300}}\\ \vdots & \vdots & \vdots \end{array}\\ \begin{array}{ccc} {\tilde{x}}_{2_{1}} & {\tilde{y}}_{2_{1}} & {\tilde{z}}_{2_{1}}\\ \vdots & \vdots & \vdots \\ \vdots & \vdots & \vdots \end{array}\\ \begin{array}{ccc} {\tilde{x}}_{15_{300}} & {\tilde{y}}_{15_{300}} & {\tilde{z}}_{15_{300}} \end{array}\\ \begin{array}{ccc} \vdots & \vdots & \vdots \end{array}\\ \begin{array}{ccc} {\tilde{x}}_{15_{1}} & {\tilde{y}}_{15_{1}} & {\tilde{z}}_{15_{1}} \end{array} \end{array}\rceil$$

Connecting the time series constituted adding additional time series from the individuals. The even indexes were flipped to create the loop for the signal. After applying the above steps, the new coordinates (*x*, *y*, *z*) for LKNE and CoM were 4500 points long. The quantiles’ function was then applied. This function returned the quantiles of the input data elements for probabilities in the range [0.1, 1]. This procedure reduced the impulsive peaks in the output trajectories, resulting in shorter time series (4048 points).

#### 2.3.2. Hurst Exponent

One of the most common tools to measure autocorrelation (persistence and long memory) and the level of noise is the Hurst exponent. The Hurst exponent (H) is defined as follows:

$${\left(R/S\right)}_{n}=C\cdot n^{H}$$

where $\left(R/S\right)$—rescaled range; *R*—range of *n* cumulative deviations from the average; *S*—the standard deviation of *n* observations; *n*—the number of elements of the time series; C—positive constant, and H—Hurst exponent. To calculate the Hurst exponent, it is necessary to compute the mean value of ${\left(R/S\right)}_{n}$ for different *n*, and then, using ordinary linear regression, determine *H* from the equation:

$$logE{\left(R/S\right)}_{n}=Hlog\left(n\right)+log\left(c\right)$$

where $E{\left(R/S\right)}_{n}$—expected value of the rescaled range [20].

Due to the specific nature of the behavior that occurs at certain values of the exponent, it was possible to determine three ranges of its occurrence: (1) 0 ≤ H < 0.5—the time series is anti-persistent, characterized by high variability; (2) H = 0.5—represents a Brownian Motion. The examined time series does not have a dominant trend by which subsequent changes take on a random character; (3) 0.5 < H ≤ 1—the time series is persistent. This time series follows an orderly course, which has the effect of maintaining the current trend. The orderliness of this interval is greater when the value of the exponent is higher [49]. In the present paper, the Kugiumtzis and Tsimpiris [50] code published on https://www.mathworks.com/matlabcentral/fileexchange/27561-measures-of-analysis-of-time-series-toolkit-mats (accessed on 20 June 2022) was applied for LKNEx, LKNEy, LKNEz, and CoMx, CoMy, CoMz time series for jazz and classic pirouettes constructed as described in the previous section.

#### 2.3.3. Test of Non-Stationarity

A time series is non-stationary if its statistical structure changes over time. The code of Zhivomirov and Nedelchev [51] was applied to check the signal’s non-stationarity (https://www.mathworks.com/matlabcentral/fileexchange/75118-signal-stationarity-estimation-with-matlab) (accessed on 2 June 2022). The new CoM and LKNE time series for all x, y, and z coordinates, for both pirouettes, were non-stationary.

#### 2.3.4. Nonlinearity of Time Series

The surrogate data test follows a null hypothesis that the signal is a realization of a linear Gaussian stochastic process (the data are a random series). Here, a surrogate data set is generated by transforming the analyzed data in a way so as to keep any characteristics recorded in the hypothesis but destroy any possible nonlinear features in the data. The iterative amplitude-adjusted Fourier transform (IAAFT) method was applied to generate the surrogate data [52]. New data matched the amplitude spectrum and signal distribution, according to Schreiber and Schmitz [53]. Then, one or more test statistics from the distribution of the surrogates and the original series were calculated. If the values of the test statistics differ significantly, the null hypothesis is rejected, suggesting that the series under consideration has a deterministic character. Here, the time series were nonlinear.

#### 2.3.5. Detection of Chaos Based on Largest Lyapunov Exponent

Using the Largest Lyapunov exponent (*LyE*) to identify chaos in a system assumes that, if the average distance between two points increases exponentially, then the system is sensitive to a change in the initial conditions (the value of *LyE* is higher than zero). Thus, *LyE* can be described by the equation: $d\left(t\right)=Ce^{LyEt}$, where *d(t)* is the average divergence at time *t,* and *C* is a constant that normalizes the initial separation. Therefore, a positive *LyE* is considered a necessary and sufficient condition for the presence of chaos in the system. For stable boundary cycles, the Lyapunov exponent is zero, and for stable equilibrium points, it is negative [54].

In this paper, the largest Lyapunov exponent was computed for new time series using the lyapunovExponent function, which is available in MatLab. This algorithm is based on the paper by Rosenstein, Collins, and De Luca [16]. According to the MatLab website, the embedding dimension (D) and time delay (tau) values were input into the lyapunovExponent function, the calculation of which is described in the next section.

#### 2.3.6. Embedding Dimension, Time Delay, and Phase Space Reconstruction

The embedding dimension (D) and time delay (tau) are key factors in phase space reconstruction. First, the time delay was estimated using the first minimum of the average mutual information function (AMIF), as discussed in [55]. For this purpose, the mdDelay function was used as follows:

tau = mdDelay(LKNE or CoM data, ‘maxLag’, 100, ‘plottype’, ‘all’).

In this code, the maximum time delay was set to 100 using the ‘maxLag’ parameter. The default value of 10 was not large enough, as shown by the output data in Figure 4 (in the Results section, the mutual information did not reach the minimum for those delays less than or equal to 10). The ‘plottype’ parameter was set to “all”, which means that the AMIF for each data dimension is presented in the plot. The mdDelay function used the threshold (1/e) criterion per default [55].

In the next step, the embedding dimension (D) was determined using the false nearest neighbors (FNN) function, which computes the percentage of false nearest neighbors for multidimensional input time series as a function of the embedding dimension [28]. To estimate the embedding dimension, the mdFnn function was used:

[fnnPerc, embTimes] = mdFnn(LKNE or CoM data, tau); where tau is the time lag calculated in the previous step for a given time series.

The functions presented above are well described in the paper by Wallot and Mønster [55] and are available on github.com/danm0nster/mdembedding (accessed on 20 June 2022). It is worth noting that the LKNE and CoM points are matrices constructed from x, y, and z coordinates describing the position of the knee joint and the center of mass for jazz and classic pirouettes. Therefore, from this point of view, they are dependent variables. The embedding dimension and time lag were calculated for LKNE and CoM for 3D space (named global), as for the Lorenz system [55,56], and also for each coordinate separately (named directional).

A total of 12 attractors were reconstructed after applying global and directional tau and D for the LKNE and CoM coordinates. Then, convex hulls were determined for each time series [57]. The convex hull of a sample of points is the minimum convex set enclosing them all [58]. Calculating the volume of the convex hull made it possible to compare the phase spaces [59]. The phase spaces were compared within the coordinates (x, y, z) of the LKNE and CoM points and between the pirouettes.

### 2.4. Recurrence Quantification Analysis (RQA)

Recurrence quantification analysis (RQA) was applied to quantify the duration and number of recurrences [5,60] presented within the generated state space for CoM and LKNE, separately for x, y, and z directions, for each pirouette. As was carried out previously, the analysis was performed for global and directional time lags and embedding dimensions. The recurrence plot is symmetrical with respect to the main diagonal. Thus, all quantitative features take place within the upper triangle. The main diagonal and the lower triangle (which provides only redundant information) are excluded from the analysis [61].

This paper uses the Toolbox of recurrence plot and recurrence quantification analysis (https://www.mathworks.com/matlabcentral/fileexchange/58246-tool-box-of-recurrence-plot-and-recurrence-quantification-analysis) (accessed on 2 June 2022) [62]. A good description of the introduced tool can be found in [63,64].

In this paper, six measures have been calculated as follows: % of recurrence (REC), % of determinism (DET), the length of the longest diagonal line segment in the plot (LMAX), Shannon information entropy (ENT), % of laminarity (LAM), trapping time (TT) [12]. The listed measures are the result of the density of the recurrence points and the structure of the diagonal and vertical lines of the recurrence plot.

The *REC* describes the percentage of recurrent points lying within a specified radius *r*(*i*). This variable can range from 0% (no recurrent points) to 100% (all points recurrent) [12,65].

$$REC=\frac{1}{N^{2}}\sum_{i,j=1}^{N}R\left(i,j\right);$$

where $R\left(i,j\right)=\Theta \left(r\left(i\right)-\|x\left(i\right)-x\left(j\right)\|\right)$; *i, j* = *1, 2, …, N*; *N*—number of points on the phase space trajectory; $r\left(i\right)$—specified threshold, ‖‖—the norm or metric (In this study, the Euclidean norm was calculated), $\Theta \left(\right)$—the Heaviside-function, defined as:

$$\Theta \left(x\right)=\left\{\begin{array}{c} 1,\;x\geq 0\\ 0,\;x<0 \end{array}\right.$$

The 0 and 1 values of the Heaviside function are represented in white and black, respectively. According to Zbilut, et al. [66], the *r*(*i*) value was set at 1% of the maximum phase space diameter.

*DET* determines the proportion of recurrent points lying along diagonal line structures, except those within the main diagonal [61]. The diagonal line segments must have a certain minimum length to be not excluded. In this paper, according to Riley, et al. [67], the choice of 2 points was considered as the number of successive points defining a line segment.

$$DET=\frac{\sum_{l=l_{min}}^{N}lP\left(l\right)}{\sum_{i=1}^{N}lP\left(l\right)};$$

where $P\left(l\right)$—histogram of the lengths l of the diagonal lines; N—number of points on the phase space trajectory, $l_{min}=2.$

*LMAX* is the length of the longest diagonal line in the plot, excluding the main diagonal. The lower the *LMAX* value, the more chaotic the analyzed signal is [12,65].

$$LMAX=max\left(\left\{l_{i};i=1,\ldots N_{l}\right\}\right);$$

where $l_{i}$—diagonal structures; $N_{l}$—number of diagonal lines in the recurrence plot.

*ENT* is the Shannon entropy of the probability $p\left(l\right)=\frac{P\left(l\right)}{N_{l}}$ to find a diagonal line of length l in the recurrence plot among the total number of diagonal lines $N_{l}$ [12,65]. *ENT* demonstrates the complexity of the recurrent trajectories versus the diagonal lines.

$$ENT=-\sum_{l=l_{min}}^{N}p\left(l\right)lnp\left(l\right).$$

*LAM* is similar to *DET*. *LAM* measures the percentage of repeated points containing vertical line structures, not diagonal lines like in the case of *DET*. *TT* is simply the average length of vertical line structures [12,60,65]. For *LAM* and *TT*, the value of the minimum line was 2 points, as for *DET*.

$$LAM=\frac{\sum_{v=v_{min}}^{N}vP\left(v\right)}{\sum_{v=1}^{N}vP\left(v\right)};\;TT=\frac{\sum_{v=v_{min}}^{N}vP\left(v\right)}{\sum_{v=v_{min}}^{N}P\left(v\right)};$$

where $P\left(v\right)$—is the histogram of the lengths of the vertical lines, N—number of points on the phase space trajectory.

## 3. Results

### 3.1. New Time Series Reconstruction

**Figure 2:**
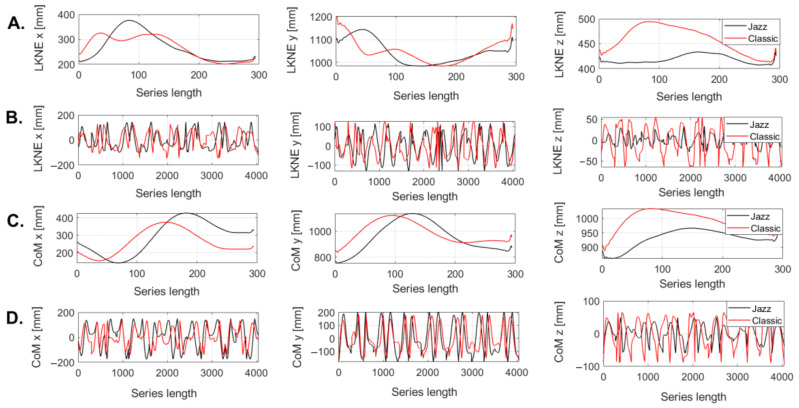
Time series for LKNE marker and CoM for x, y, and z coordinates for jazz and classic pirouettes (**A**,**C**) for a single individual after the resampling procedure; (**B**,**D**) reconstruction of the time series before applying the quantiles function.

The reconstruction of the new time series proceeded for each of the x, y, and z coordinate of the LKNE marker and CoM for the jazz and classic pirouette, respectively. The recorded time series were short (101 to 250 points). Therefore, for each person, the series were resampled to 300 points (Figure 2A,C). Figure 2A,C show the LKNE and CoM curves for a single person for two pirouettes to help understand the creation of a new series and demonstrate their structure (Figure 2B,D). Importantly, all further calculations involved new time series.

### 3.2. Hurst Exponent Analysis and the Largest Lyapunov Exponent

The Hurst exponent was calculated to check if the new time series showed persistence and robustness and to find if there was any significant difference between the two pirouettes. The time series for the coordinates (x, y, z) of LKNE and CoM had H values of between 0.5 and 1, implying that the time series were persistent (Table 3). On average, for both pirouettes, the H values were 0.72.

The values of the largest Lyapunov exponents, which were positive and indicate the presence of chaos for the analyzed time series, are shown in Table 4. The average LyE is 1.64 and 1.78 for the classic and jazz pirouettes, respectively.

### 3.3. Determination of the Embedding Dimension and Time Delay

The global embedding dimension, calculated in 3D space (all D), was 2 for each time series (CoM and LKNE) except for the LKNE from the jazz pirouette, for which this value was equal to 3.

**Figure 3:**
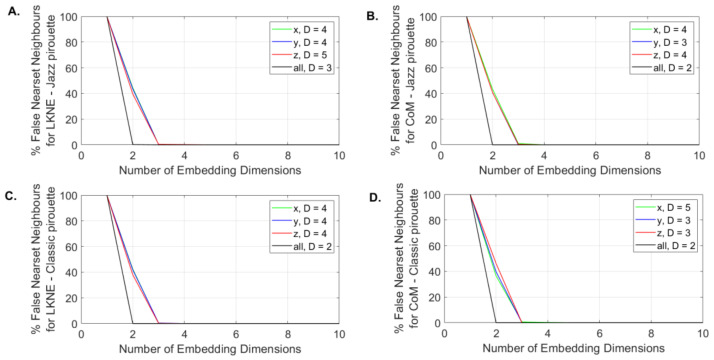
The global (all D) and directional (xD, yD, zD) embedding dimensions as an output of the mdFnn function for the (**A**) LKNE marker for jazz pirouettes; (**B**) CoM for jazz pirouettes; (**C**) LKNE marker for classic pirouettes, and (**D**) CoM for classic pirouettes.

The directional embedding dimension calculated for the knee of the supported limb was 4 for the jazz and classic pirouettes in the x and y directions (Figure 3A,C). For the z-direction, the dimension was 1 greater for the jazz pirouette (Figure 3A,C). For CoM, the identical embedding dimension was found only in the y-direction (D = 3) (Figure 3B,D).

**Figure 4:**
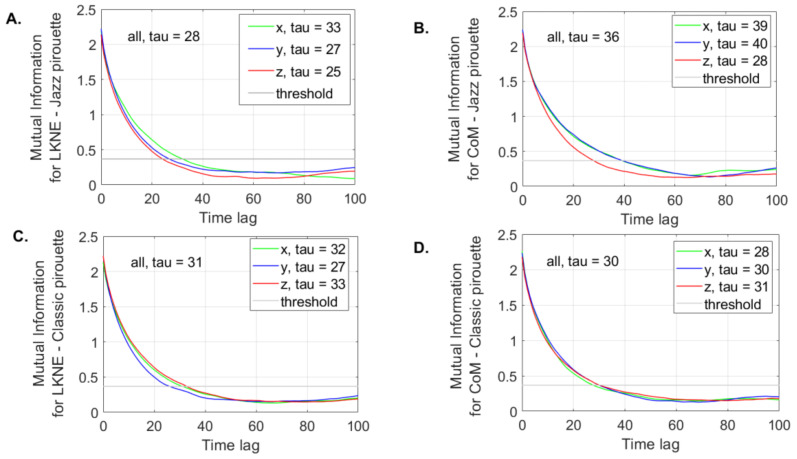
The global (all tau) and directional (xtau, ytau, ztau) time delays as the output of the mdDelay function for (**A**) LKNE marker for jazz pirouette; (**B**) CoM for jazz pirouette; (**C**) LKNE marker for classic pirouette; (**D**) CoM for classic pirouette. The threshold was set at 1/e.

The global mutual information (all tau) was the average of the minimum values calculated separately for the LKNE and CoM (x, y, z) time series (Figure 4).

For the LKNE time series of the classic pirouette, the all tau value increased by 11% compared to the value calculated for the jazz pirouette (Figure 4A,C). For CoM, the situation was the opposite. The all tau value for the classic pirouette decreased by 16% compared to the value noted for jazz (Figure 4B,D).

### 3.4. Phases Space Reconstruction and Convex Hull Calculation

**Figure 5:**
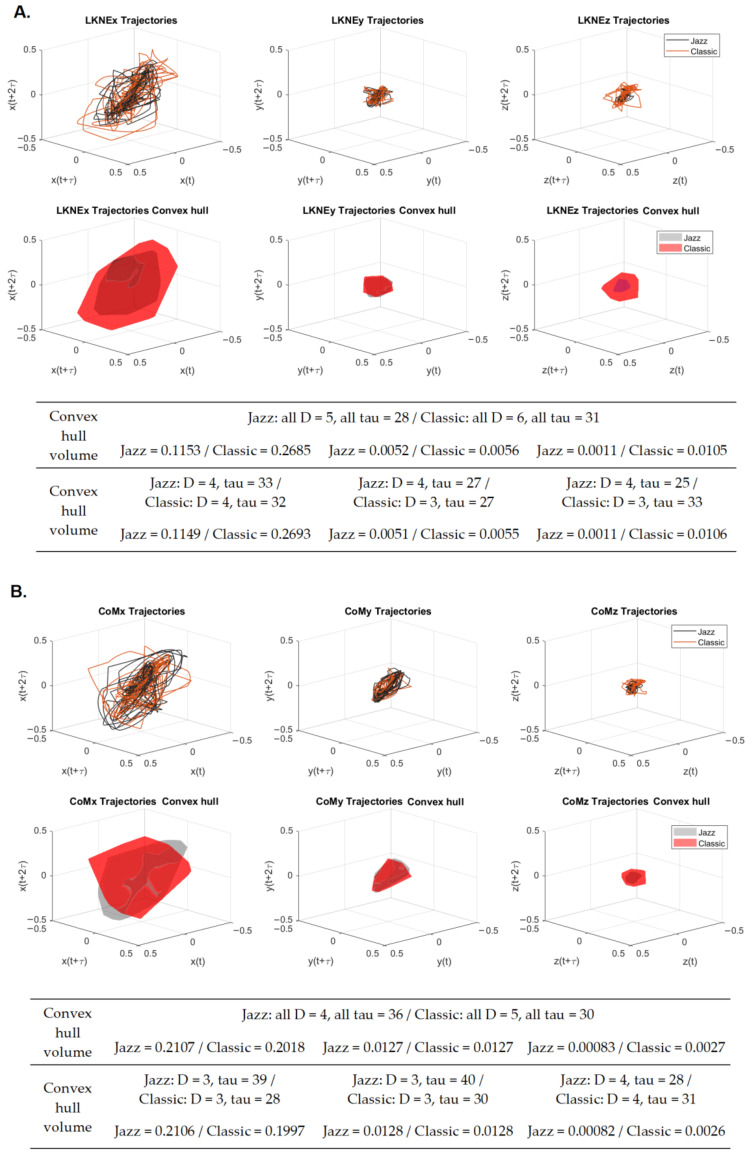
The reconstructed phase space (odd lines) with a global time lag (τ = all tau), convex hulls, and their volumes (even lines) for the (**A**) LKNE marker and (**B**) CoM x, y, and z coordinates for jazz and classic pirouettes (x-anterior-posterior, y-inferior-superior, z-mediolateral). The tables show convex hull volumes for global and directional parameters (D and tau).

The reconstructed 3D phase space for LKNE and CoM in a group of 15 dancers was treated as a long time series (Figure 5). The reconstruction was carried out using the global embedding dimension (all D) and global time delay (all tau) (Figure 3 and Figure 4).

In addition, phase space reconstruction was also performed using embedding dimensions and time lags specific to the x, y, and z directions of the analyzed time series. Since the visualization of the phase space reconstruction had not changed, only the values of the convex hull volumes (calculated for the global tau and D and those directional ones) are included in Figure 5. It is worth highlighting that the two reconstruction methods yielded similar results.

The convex hull values calculated in both ways for the LKNE were always significantly higher for the classic pirouette than for the jazz pirouette (Figure 5A). The highest percentage differences were sequentially for LKNEz (global tau and D—854.55%, directional tau and D–864.51%), which are the mediolateral motion, LKNEx (anterior-posterior), with 132.87% (134.38%), and LKNEy (inferior-superior), with 7.69% (7.84%).

For the CoM, the situation was different (Figure 5B). The trend of the changes continued only for the z-direction (mediolateral), where the convex hull volume value was 225.3% (217.07%), which was higher for the classic pirouette. For the y-direction (inferior-superior), the volume values were equal. However, for the x-direction (anterior-posterior), the convex hull volume value was 4.41% (5.46%), which was higher for the jazz pirouette. Therefore, the classic pirouette appears to be the most compact for CoM x and y, as the convex hull volumes are the smallest in these directions. The jazz pirouette is more coherent for the knee joint. The knee joint for this pirouette has a slight change in the range of motion during rotation compared to the initial and final phases.

### 3.5. Recurrence Quantification Analysis

Recurrence is a measure of the reproducibility of a limit cycle. The recurrence values were calculated for global tau and D and for directional ones. The results are shown in Table 5.

It is worth noting that only the REC values calculated for the LKNE marker for the jazz pirouette in each direction were higher (by an average of 7.13%) regarding the global setting (Table 5). Overall, the mean percentage difference between recurrence measures calculated for the global, and directional settings were higher for the classic pirouette. DET and LAM are the parameters with the lowest changes, while the most significant differences were recorded for TT and REC (Table 6).

**Figure 6:**
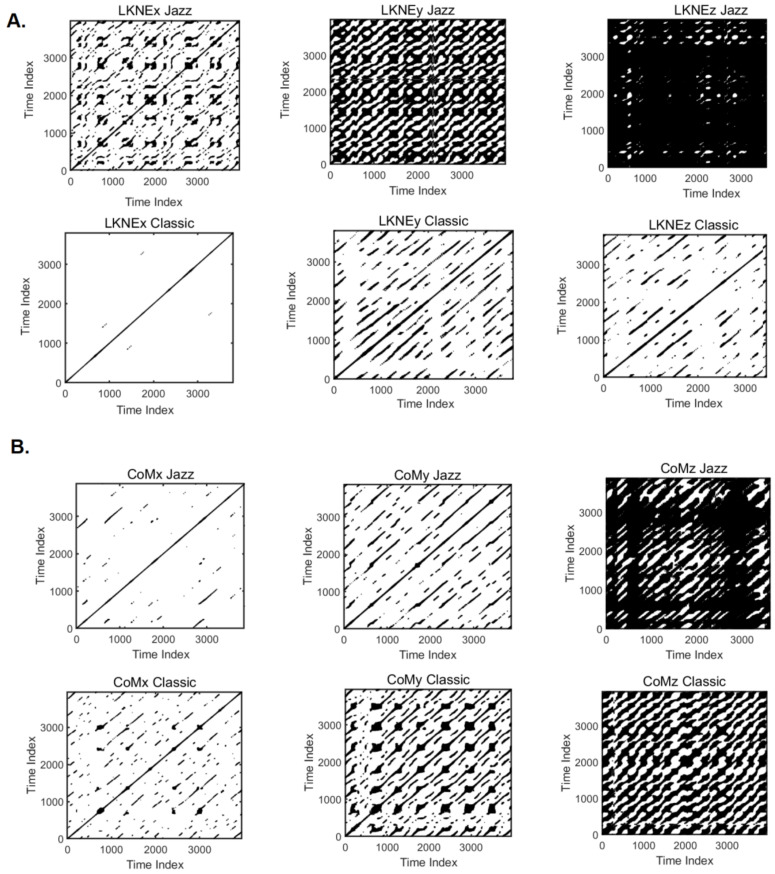
The illustration of the multiscale recurrence analysis for the (**A**) LKNE and (**B**) CoM time series separately for the x-anterior-posterior, y-inferior-superior, z-mediolateral directions for jazz and classic pirouette. Graphs obtained for the global time delay and embedding dimensions.

The shorter lengths of the diagonal lines within the recurrence plots indicate lower recurrence rates in the phase space. This is mainly evident for LKNEx, when compared to LKNEz and LKNEy in the classic pirouette (Figure 6). The highest recurrence rate (allREC = 90.41%; dREC = 82.46%) was for LKNE_z across the mediolateral axis in the jazz pirouette (Table 5). High values (allREC = 86.35%; dREC = 86.52%) were also recorded for CoM_z for the same pirouette.

The recurring points form easily visible diagonals that are parallel to the main diagonal (origin line) yet offset from it. This is typical of a periodic deterministic structure (Figure 6). The percentage determinism (DET), calculated for global and directional settings, was extremely high throughout the LKNE and CoM for all coordinates in both pirouettes (Table 5). The highest DET value was for the CoM and LKNE during the Jazz pirouette in the z-direction, similar to REC. The %LAM was similar to %DET. This measures the percentage of the repeated points containing vertical line structures rather than diagonal lines. Here, the LAM values (allLAM and dLAM) are high, showing that the vertical line structures are widespread in the analyzed pirouettes. The highest value was, as before, for CoMz and LKNEz for the jazz pirouette. TT is the average length of vertical line structures. The highest TT value was found for CoM and LKNE in the z-direction for the jazz pirouette, and the lowest was found for LKNE for the classic pirouette in the x-direction. As for entropy, its lowest values were for LKNEx, y, and z for the classic pirouette. The highest entropy value (allENT = 8.23 and dENT = 8.26) and, thus, the most complex signal was that of CoMz for the jazz pirouette. The entropy values calculated for LKNEz (allENT = 7.54 and dENT = 8.17) were also not much lower for the jazz pirouette.

## 4. Discussion

The pirouette is an essential skill for all gymnasts and dancers. It is one of the most popular movements used in training [68]. The purpose of this study was to evaluate the geometric structure of the related attractors and analyze “recurrence” in two different pirouettes (jazz and classic), as performed by professional dancers. According to Kantz and Schreiber [69], the phase space representation approach, rather than the time or frequency domain analysis, is a feature of nonlinear dynamical time series analysis. Moreover, recurrence quantification analysis (RQA) is an alternative nonlinear method for describing signal dynamics. It is remarkably robust for short, noisy, and non-stationary signals [70,71,72]. This approach was used to observe if the evaluated pirouettes in the study group were recurrent. In addition, it was possible to see which pirouette is more stable or complex.

Until now, no one has focused on attractor reconstruction for a group of dancers performing pirouettes. This study considered the most characteristic time series that differentiated the jazz and classic pirouettes. These included the center of body mass (CoM) and the position of the knee joint of the supporting limb (LKNE). The mentioned parameters are characteristic due to the manner in which the pirouettes are performed [46]. In the middle of the turn phase, the average minimum knee flexion value for the classic pirouette was 2.4°, while for the jazz pirouette, it was 22.8° [46]. In addition, Błażkiewicz [46] showed that pelvic alignment changes when performing these two pirouettes, so the positioning of the center of mass is different.

It is worth noting that typical pirouettes produce short time series, which contain about one to two revolutions. These time series are not long enough to use nonlinear measures and reconstruct the attractor, as most conditions will not be satisfied. The present study addressed this challenge by constructing a new CoM and LKNE time series, containing data from all the studied dancers. The dancers performed only single turns. It is crucial to mention that the dancers were from one dance group and had been dancing with each other for more than four years. Therefore, one may assume that the individual dancers were at the same level of motor development as far as the performance of the analyzed movements is concerned. The time series of the selected points were resampled, normalized, and randomly reordered. Then every second time series was flipped to be combined with other time series and make a long time series out of the repetitions of a single task. This approach made it possible to satisfy the conditions for the presence of chaos.

After lengthening the time series, it turned out that there are positive Lyapunov exponents in three directions for the CoM and LKNE for jazz and classic pirouettes, indicating the presence of chaos. In this study, the Rosenstein algorithm [16] was used to calculate the Lyapunov exponent. The values for LyE for CoM were within the ranges reported by Mehdizadeh [73] for the upper trunk during gait with different velocities. The ranges of LyE values reported by Mehdizadeh [73] were 0.15–3.30, 0.10–2.85, and 0.27–2.40 for anterior-posterior, mediolateral, and vertical directions, respectively. In this study, the average LyE calculated for CoM (x, y, z) was 1.62 for classic pirouettes and 1.84 for jazz pirouettes. In contrast, the average LyE calculated for LKNE (x, y, z) and CoM (x, y, z) was 1.64 and 1.78 for the classic and jazz pirouettes, respectively. Moreover, Hurst exponent analysis was applied to check the persistence of these new time series, which showed all out-coming time series to have a persistent behavior. On average, for both pirouettes, the H values were 0.72. The similar values for the Hurst exponent indicate that both pirouettes have a stable movement pattern for both the knee joint and the center of mass. It is worth highlighting that this is the first approach in the literature that presents such preparation of the time series. This preparation allows dancers to be treated as samples for the same dance moves, and thus, this time series can evaluate the global system’s dynamic behavior. Unfortunately, this approach does not allow for the individual assessment of the dancers. The global dynamic evaluation of classic and jazz pirouettes for the new time series was realized using RQA and phase space reconstruction. It is worth mentioning that the phase space reconstruction and RQA analysis were specific to embedding dimensions and time delay. Here, two types of these parameters were detected: global and directional. The directional parameters were calculated separately for the x, y, and z directions of the displacements of the analyzed points (LKNE and CoM) on the dancer’s body. However, assuming that the x, y, and z variables are dependent because they describe the movement of individual body parts, the so-called global parameters were then calculated. It is worth highlighting that both approaches yield similar results for RQA and phase space reconstruction, although the results for directional parameters were slightly higher.

The results of the RQA analysis showed that the percentage determinism (DET) calculated for both ways was exceptionally high (99.59–99.98%) for LKNE and CoM (for all coordinates) in both pirouettes. It indicates that the signals have deterministic features due to repeated (recurrent) time series at various frequencies (because they came from different individuals). A determinism of 100, meaning perfect cyclic motion, is near impossible and probably undesirable, even in professional athletes. This could be related to skill optimization in the learning process [74] and the functional variability required for flexible movement behavior [75]. The signals for the jazz pirouette had a very high recurrence. The recurrence was the highest for this pirouette along the mediolateral axis at the knee joint (REC = 90.41% (global), REC = 82.46% (directional)), and CoM (REC = 86.35% (global), REC = 86.52% (directional)). The lowest recurrence was for the x-direction of the knee in the classic pirouette (REC = 1.6% (global), REC = 1.62% (directional)). On the other hand, the entropy value for this movement was the lowest, which suggests little complexity in the distribution of periodic components [76]. The high values for the RQA measures, like trapping time (TT), laminarity (%LAM), and a maximum length of a diagonal line (LMAX), imply low complexity in the system’s dynamics. In the case of this paper, it is in the mediolateral direction for the knee joint for the jazz pirouette.

The attractor reconstruction process confirmed the results discussed for the recurrence quantification analysis. The convex hulls were calculated to compare the reconstructed attractors. According to Zhao [59], convex hulls are a common way to generalize the shape of an attractor and extract useful information, which can be used for comparison. The values for the volumes of the convex hulls were, on average, 51.41% (51.24%-calculated for directional tau and D), with this being smaller for the knee joint in the jazz pirouette compared to those calculated for the classic pirouette. This means that the jazz pirouette is more coherent for the knee joint of the supporting limb. On the other hand, for CoM, the average value of convex hulls volume was 4.06% (3.13%-calculated for directional tau and D), slightly smaller for the classic pirouette compared to Jazz. Therefore, the classic pirouette seems to be a little more compact for the CoM. The main direction causing these changes was the motion along the *x*-axis for both LKNE and CoM. The convex hull volume values were the highest for the x-direction (anterior-posterior) and lowest for the z-direction (mediolateral). Once again, referring to the paper of Błażkiewicz [46], it is worth emphasizing that the range of motion in all joints of the supporting leg in the sagittal plane, and the hip joint in the frontal plane for the non-supporting limb, was significantly higher for the classic pirouette compared to the jazz one. This makes the classic pirouette more challenging because it requires higher precision of movement, better neuromuscular control, and thus stability.

It is worth emphasizing that the discussed methodology is not popular. The author found only one paper [77] that dealt with similar issues. Burton et al. [77] evaluated long swings from the perspective of attractor dynamics. They proved that the senior gymnasts displayed increased recurrence characteristics in addition to a long, long swing duration and a lower angular velocity of their center of mass. Moreover, all groups of gymnasts had highly recurrent and predictable limit cycle characteristics. Burton et al. [77] showed that attractor reconstruction helps to understand the development of an athlete’s skills and physical preparation. However, the cited study had an adequate time series length for each participant, allowing for local and global evaluations of the system’s dynamics.

## 5. Conclusions

The approach shown in this paper made it possible to evaluate a group of dancers as a single, global organism, performing two types of pirouettes. The jazz pirouette, and more precisely, the movement of the knee joint of the supporting limb, which was assessed in the way presented in the paper, is more compact and reproducible (smaller convex hull volume values and large REC values) compared to the classic pirouette. The emergent dynamics of the dance movement, and the established similarities between the dancers, suggest that the global level of movement may somehow be disconnected from the lower-level neuromuscular dynamics that generate it. Thus, the results show that the global behavior of the pirouette, instead of the local, is individual to the behavior of the dancers. This approach has not been applied in the literature and might be controversial. Overall, the reconstruction of the attractors, the evaluation of the Lyapunov exponent and the Hurst exponent, and the application of RQA appear to provide a comprehensive view of the general motion presented by the dancers.

## Figures and Tables

**Table 1:** Methods for calculating the time delay τ (tau) and the embedding dimension (D) required for phase space reconstruction.

Phase Space Reconstruction State Vector: y(t) = (x(t), x(t + τ), x(t + 2τ), …, x(t + (D − 1)τ)) from Original Time Series Data x(t) by Using Time Delay (τ) and Dimension of the Attractor D	
Possible ways of τ selection [24]: (1) Autocorrelation function (2) Mutual information	Possible ways of D selection: (1) Principal Component Analysis (PCA) [25] (2) Correlation dimension [26] (3) Box-counting [27] (4) False Nearest Neighbor (FNN) [28]

**Table 2:** Mean and standard deviation of anthropometric parameters describing the dancers.

Group	Age [Years]	Body Mass [kg]	Body Height [m]	Training Period [Years]
*N* = 15	22.13 ± 2.73	57.56 ± 6.76	1.68 ± 0.62	12.19 ± 3.04

**Table 3:** The Hurst exponent values calculated for the x, y, and z coordinates of the reconstructed LKNE and CoM time series for classic and jazz pirouettes.

	LKNEx	LKNEy	LKNEz	CoMx	CoMy	CoMz
**Classic**	0.75	0.71	0.71	0.75	0.72	0.70
**Jazz**	0.76	0.66	0.70	0.73	0.69	0.75

**Table 4:** The Lyapunov exponent values calculated for the x, y, and z coordinates of the reconstructed LKNE and CoM time series for classic and jazz pirouettes (for the directional dimensions and lags from Figure 3 and Figure 4).

	LKNEx	LKNEy	LKNEz	CoMx	CoMy	CoMz
**Classic**	1.53	1.43	2.05	2.11	1.44	1.3
**Jazz**	1.43	1.13	2.57	2.04	1.79	1.69

**Table 5:** Recurrence variables calculated for global and directional time lag and the embedding dimension for the LKNE marker and CoM motion in x-, y-, and z-directions for J—jazz and C—classic pirouettes. REC—% of recurrence, DET—%determinism, LMAX—the length of the longest diagonal line segment in the plot, ENT—Shannon information entropy, LAM—%laminarity, TT—trapping time. The prefix ‘all’ means global, and d denotes directional.

	allREC	dREC	allDET	dDET	allLMAX	dLMAX	allENT	dENT	allLAM	dLAM	allTT	dTT
**LKNEx_J**	8.61	8.08	99.59	99.60	1018	1019	5.75	5.83	99.74	99.77	16.54	19.95
**LKNEy_J**	43.34	41.2	99.8	99.85	2212	2243	6.9	7.1	99.9	99.93	54.08	65.31
**LKNEz_J**	90.41	82.46	99.93	99.96	3935	3972	7.54	8.17	99.97	99.98	163.39	223.12
**LKNEx_C**	1.6	1.62	99.6	99.61	1246	1305	5.69	5.75	98.33	99.65	9.7	15.36
**LKNEy_C**	16.3	20.1	99.72	99.86	2109	2210	6.46	7.05	99.87	99.94	43.45	77.38
**LKNEz_C**	10.5	18.51	99.81	99.92	2425	2514	6.44	7.23	99.9	99.97	36.36	67.22
**CoMx_J**	2	2.87	99.73	99.76	915	945	6.29	6.30	99.74	99.85	16.2	20.82
**CoMy_J**	11.97	18.16	99.91	99.93	3939	3967	7.23	7.37	99.95	99.97	37.3	46.92
**CoMz_J**	86.35	86.52	99.98	99.98	3939	3963	8.23	8.26	99.99	99.99	261.59	262
**CoMx_C**	1.12	1.63	99.65	99.74	1536	1600	6.25	6.32	99.64	99.85	12.36	20.96
**CoMy_C**	9.84	12.74	99.87	99.93	3927	3987	6.83	7.14	99.95	99.98	38.04	55.04
**CoMz_C**	32.59	42.18	99.93	99.94	3927	3954	7.15	7.32	99.97	99.98	76.2	89.77

**Table 6:** The mean percentage difference between recurrence measures calculated for the global and directional settings.

	REC	DET	LMAX	ENT	LAM	TT
**Jazz**	19.47	0.02	1.17	2.52	0.03	22.07
**Classic**	34.22	0.07	3.27	5.08	0.29	58.9

## Data Availability

The measurement data used to support the findings of this study are available from the corresponding author upon request.
